# Inadequate Minority Representation within SARS-CoV-2 Vaccine Trials

**DOI:** 10.4269/ajtmh.20-1294

**Published:** 2020-11-11

**Authors:** Joshua F. Craft, Mark A. Travassos, Carlo Foppiano Palacios, John J. Openshaw

**Affiliations:** 1 Center for Vaccine Development and Global Health, University of Maryland School of Medicine, Baltimore, Maryland;; 2 Department of Internal Medicine, Infectious Diseases Section, Yale School of Medicine, New Haven, Connecticut;; 3 Division of Infectious Diseases and Geographic Medicine, Stanford University, Stanford, California

## Abstract

Minority communities have borne the brunt of COVID-19 disease in the United States. Nonwhites have contracted most of the SARS-CoV-2 infections; COVID-19 mortality rates for Black Americans are more than twice those for whites. Given this, studying the most effective ways to prevent and treat SARS-CoV-2 in these populations should be a research priority, particularly with respect to vaccine trials. Federal guidelines from the National Institutes of Health and Food and Drug Administration emphasize the need for inclusion of minority groups in these trials, but none of the publicly available SARS-CoV-2 vaccine trial protocols requires representative sampling of minorities. This piece emphasizes the importance of adequate inclusion of minority communities in SARS-CoV-2 vaccine trials, and the implications of this inclusion for SARS-CoV-2 vaccine distribution.

Minority communities, namely, Black, Latinx, and Indigenous (Native American/Alaska Native), have borne the brunt of COVID-19 disease in the United States. Minorities have contracted most of the SARS-CoV-2 infections in the United States, with 33% of all infections occurring in Latinx individuals and 22% in Black individuals.^1^ In addition, the COVID-19 mortality rate for Blacks is more than twice that of whites, and mortality rates among Latinx and Indigenous peoples are almost 50% higher than those of whites.^2^

As the medical community undertakes efforts to reduce COVID prevalence, morbidity, and mortality, we must sharpen our focus on these most affected communities. Historically, when considering allocation of limited vaccine supply, national efforts have prioritized the most vulnerable populations. In the 1950s, the U.S. Public Health Service prioritized children aged 5–9 years to receive the new polio vaccine.^3^ In the 2009 H1N1 influenza epidemic, the CDC’s Advisory Committee on Immunization Practices recommended that the most vulnerable U.S. populations, including pregnant women, children, and immunocompromised adults or those with chronic health issues, be first to receive immunization.^4^ Following these historical precedents, for a SARS-CoV-2 vaccine to have the maximal impact, it must be safe, effective, and accepted in today’s most vulnerable populations. To ensure this, trials today must systematically enroll Black, Latinx, and Indigenous individuals at levels proportional to the general population at minimum, and ideally at levels proportional to disease prevalence. If ongoing and future vaccine trials do not represent these minority populations, we will be jeopardizing the safety of these vulnerable communities and providing a vaccine of uncertain safety and efficacy.

Just as important as developing a safe and effective SARS-CoV-2 vaccine, the medical community must also convince the public of the benefits of receiving this vaccine. Some members of the medical community have spoken out about the need for greater minority inclusion in SARS-CoV-2 vaccine trials,^5^ and this issue has received attention in local newspapers, national media outlets across the political spectrum, and international news agencies. Underlying this focus is the fact that, even at this historic moment, there is an unprecedented level of distrust in medical expertise. Within Black and Latinx communities particularly,^6^ this distrust is long-standing and based on the medical community’s legacy of racist practices. Scholars have documented and analyzed the origins, history, and modern legacy of racist medical practices in the United States,^7,8^ but the medical community at large has yet to act upon the lessons contained in this body of literature. Perhaps the most widely known example of medical racism in the United States, and the most egregious in scope, is the U.S. Public Health Service Tuskegee Syphilis Study, which permitted syphilis to go untreated for over four decades in hundreds of Black American men.^9^ Medical racism in the United States has even appropriated the bodies of its victims. In 1951, researchers at the Johns Hopkins University School of Medicine developed an immortal cell line from the body of Henrietta Lacks, a 31-year-old Black woman with cervical cancer, without her consent.^10^ Since then, the use of her cell line has become widespread throughout medicine, including in the development of Jonas Salk’s polio vaccine,^11^ raising the question of whether the medical community believes that therapeutic ends can justify racist means.

If a SARS-CoV-2 vaccine is to effectively reduce COVID morbidity and mortality, it must be accepted in the most affected and most vulnerable populations. The medical community, therefore, must sustain authentic engagement with these communities to ensure uptake of the licensed vaccine, or else we risk again marginalizing Black, Latinx, and Indigenous communities in their times of need. Actively offering enrollment to members of these communities in vaccine trials is a critical step to do this.

Pharmaceutical companies, in response to public demand for transparency, have taken the unprecedented step of making their SARS-CoV-2 vaccine trial protocols publicly available. These protocols, however, offer little reassurance to skeptical Black, Latinx, and Indigenous individuals. The NIH policy states that for diseases such as COVID-19, where it is unknown if there are vaccine efficacy differences for particular ethnic groups, a phase 3 clinical trial should be required to include “sufficient and appropriate entry of…racial/ethnic participants.”^12^ The FDA recommends, but does not require, “the enrollment of populations most affected by COVID-19, specifically racial and ethnic minorities.”^13^ Given these federal policies, it is discouraging that none of the publicly available protocols—Moderna,^14^ Pfizer,^15^ AstraZeneca,^16^ and Johnson & Johnson^17^—includes either the requirement or recommendation for proportional representation of the most affected minority communities. The Moderna protocol states that investigators seek to enroll participants “whose locations or circumstances put them at appreciable risk,”^14^ whereas the Johnson & Johnson protocol states that “Efforts will be made to ensure good representation in terms of race, ethnicity, and gender.”^17^ But none of the four protocols explicitly connect COVID-19 risk with race, despite the overwhelming data necessitating this connection be acknowledged and acted upon. Moderna’s protocol goes the furthest, stating an “aim to enroll a representative sample of participants from these minority population (sic),”^14^ but stops short of defining either “representative sample” or “minority population.” Moderna has reported that 28% of the individuals enrolled for its mRNA vaccine trial thus far are from “diverse communities,”^18^ but provides no further information on the racial composition of this group.

Adequate enrollment of Black, Latinx, and Indigenous individuals is crucial to identifying factors that may affect vaccine efficacy and safety in these populations. Although the social construct of race remains a controversial biologic concept without a clear physiologic basis, self-defined race has played a role in some outcomes in medicine, suggesting the potential role of underlying biologic mechanisms.^19^ In Indigenous North American pediatric populations, which have historically had higher rates of invasive *Haemophilus influenzae* type b disease than children in other racial groups, immunization with a conjugate Hib vaccine featuring the outer membrane protein complex has been more effective than with other Hib vaccine formulations.^20^ Thus, beyond the importance of racial representation for downstream SARS-CoV-2 vaccine uptake, there is also a need to account for race when measuring SARS-CoV-2 vaccine efficacy and safety, which could be influenced by potential unknown, race-, or ethnicity-associated biologic mechanisms. No subgroup analyses are planned to study SARS-CoV-2 vaccine safety and efficacy within these populations; and without mandated levels of enrollment specifically for enrolling Black, Latinx, or Indigenous individuals, it is possible that sample sizes will be insufficient for such analyses. By not requiring sufficient minority representation in these historic trials, the medical community runs the risk of perpetuating the disturbing racial and structural inequities evidenced thus far in the COVID-19 pandemic. By addressing this issue, the medical community would be making a commitment both to the protection of and investment in minority communities.

Black, Latinx, and Indigenous communities are most affected by COVID-19, based on the prevalence and mortality rate, so a public vaccine implementation strategy ought to prioritize these communities. If individuals from these communities are not included in trials, the medical community cannot ensure the efficacy and safety of the vaccine in these individuals. If Black, Latinx, and Indigenous individuals are not included in trials, we will be silently and forcefully marginalizing these communities and, consciously or not, furthering centuries of racist practices and mistrust. We can and must do better. By incorporating the contemporary public health data showing Black, Latinx, and Indigenous communities are disproportionately affected by and vulnerable to COVID-19 into our historical practice of directing national vaccination efforts toward the most vulnerable communities, we can create a more safe, effective, and equitable COVID-19 response. Development of a COVID-19 vaccine provides an opportunity to reimagine a medical paradigm that for too long has perpetuated racial disparities and to proactively address disease burdens in the most impacted communities.

On a global scale, the COVID-19 Vaccine Global Access Facility (COVAX) network attempts to create equity in COVID-19 treatment and prevention, bringing together a racially and economically diverse coalition of nations with the explicit goal of equitable distribution of COVID therapies.^21^ The network includes participation from a racially and economically diverse coalition of more than 180 nations and aims to create a platform to ensure that a safe and effective SARS-CoV-2 vaccine will be available to its members. By describing and starting to address the ethical issues that must be surmounted to achieve equitable SARS-CoV-2 vaccine access and distribution, COVAX is a step in the right direction in earning the trust of historically oppressed communities and championing the common interest of improved public health globally. The United States has declined to join COVAX, foregoing a valuable opportunity to address inequity within its own borders and abroad.^22^

We call upon professional medical societies such as the American Society of Tropical Medicine and Hygiene, American Medical Association, and Infectious Diseases Society of America to support this critical initiative of adequate inclusion of minority communities in SARS-CoV-2 vaccine trials. To truly acknowledge that Black, Latinx, and Indigenous lives matter in the midst of a pandemic that is killing them at a rate twice that of other Americans, we must incorporate explicit mechanisms for minority inclusion into COVID-19 vaccine trials, analysis, and early vaccine distribution.
